# Electromechanical Response and Residual Thermal Stress of Metal-Core Piezoelectric Fiber /Al Matrix Composites ^†^

**DOI:** 10.3390/s20205799

**Published:** 2020-10-13

**Authors:** Yinli Wang, Tetsuro Yanaseko, Hiroki Kurita, Hiroshi Sato, Hiroshi Asanuma, Fumio Narita

**Affiliations:** 1 Department of Materials Processing, Graduate School of Engineering, Tohoku University, Sendai 980-8579, Japan; wang.yinli.r2@dc.tohoku.ac.jp; 2 Department of Mechanical Engineering, Kogakuin University, Tokyo 192-0015, Japan; yanaseko@cc.kogakuin.ac.jp; 3 Department of Frontier Sciences for Advanced Environment, Graduate School of Environmental Studies, Tohoku University, Sendai 980-8579, Japan; kurita@material.tohoku.ac.jp; 4 Advanced Manufacturing Research Institute, National Institute of Advanced Industrial Science and Technology, Ibaraki 302-8564, Japan; h-sato@aist.go.jp; 5 Department of Mechanical Engineering, Chiba University, Chiba 263-8522, Japan; asanuma@faculty.chiba-u.jp

**Keywords:** piezoelectricity, finite element simulation, composite materials, residual stress, sensor

## Abstract

It is well known that the curing residual stress induced during a fabrication process has a great influence on the performance of piezoelectric composite devices. The purpose of this work was to evaluate the residual thermal stress of lead zirconate titanate piezoelectric fiber aluminum (Al) matrix (piezoelectric fiber/Al) composites generated during fabrication numerically and experimentally and to understand the effect of the residual thermal stress on the electromechanical response. The three-dimensional finite element method was employed, and the residual stress generated during the solidification process of the Al matrix was calculated. The output voltage was also calculated in the analysis when putting stresses on the composite materials in the length direction of the piezoelectric fiber. It was shown that the cooling from higher temperatures increases the electromechanical conversion capability. Furthermore, we also performed the simulation, and we recorded the output voltage under concentrated load to investigate its application as a load position detection sensor, and we also discussed the influence of the position by changing the modeling with a different fiber position in the Al. The residual stress of hot press molded piezoelectric fiber/Al composite was then measured, and the comparison was made with the calculated values. The simulation results revealed that our model predictions reproduced and explained the experimental observations of curing residual stress. After this study, similar models of composite materials can be analyzed by this simulation, and the result can be used to design piezoelectric composite materials.

## 1. Introduction

In recent years, as humans recognized the importance of sustainable energy, increasing attention has been paid to piezoelectric materials for their applications such as in sensors, actuators, and energy-harvesting devices. The recent advances in piezoelectric composite materials for energy harvesting applications have been summarized [1,2]. Lead zirconate titanate (PZT) is a widely used piezoelectric ceramic material; a distinctive feature of PZT is its large piezoelectricity; however, poor mechanical properties limit its fields of application. The development of piezoelectric composites helps to overcome some of the limitations of conventional PZT ceramics by improving their demerits, especially brittleness, lack of reliability and conformability [3,4,5]. 

Lead nickel niobite (PNN) and PZT particles dispersed in epoxy matrix (PNN–PZT/epoxy) have been fabricated as paint sensors on account of their low cost and weight [6], and the impact sensitivity of the sensor according to different poling conditions has been measured in order to determine the optimal conditions of poling time and electric field. Experimental studies have revealed the effect of the poling conditions on the piezoelectricity of barium titanate (BTO) particles dispersed in an epoxy matrix (BTO/epoxy) [7] and potassium sodium niobite (KNN) particles dispersed in an epoxy matrix (KNN/epoxy) [8]. Piezoelectric glass fiber-reinforced polymer (GFRP) has also been manufactured using the mixture of PNN–PZT piezoelectric powder and epoxy as a smart resin, and it was shown that the piezoelectric GFRP could be utilized as an impact sensor [9]. The polyvinylidene fluoride (PVDF) composite consisting of two PVDF films sandwiching a GFRP laminate has been proposed, and the energy-harvesting capability and open circuit voltage have been increased remarkably [10]. Compared with conventional methods, a novel material-processing technique in which PZTs are covered by a thin patch of woven E-glass fiber fabric for enhanced adhesion with the surrounding epoxy matrix was designed, and the functionality of the proposed smart composite and its sensitivity in detecting material damage were examined [11]. A macro fiber piezoelectric energy harvester has been integrated onto a carbon fiber-reinforced polymer (CFRP) composite laminate using the co-curing method [12], whereas piezoelectric CFRP composite laminate has been developed by embedding a lead-free piezoelectric nanoparticle-filled epoxy interlayer with GFRPs [13]. KNN nanoparticle-filled epoxy/CFRP composite generators have been fabricated and polarized successfully, proving that polarization was obtainable even when CFRP was used as the electrodes of KNN nanoparticle-filled epoxy resin [14]. 

On the other hand, an impact detection system based on a metal core piezoelectric fiber/aluminum (Al) composite has been developed [15]. Impact load position was estimated and the minimum required accuracy for a structural health monitoring (SHM) system was satisfied. When the piezoelectric ceramic fiber/Al matrix composite was subjected to temperature changes during its manufacturing process, differences between the coefficients of thermal expansion (CTEs) of the fiber and matrix cause large thermal stresses to be induced within the fiber. The residual thermal stresses may change the characteristics of piezoelectric composite materials. It is therefore important to accurately evaluate the magnitudes of the residual thermal stresses in the piezoelectric composites during manufacturing and their piezoelectric response during service. For PZT ceramics, it has been reported that the materials lose their piezoelectric properties near the Curie temperature; however, their domain configurations remain unchanged [16]. Algueró et al. [17] investigated the electromechanical properties of PZT ceramics by heating. The experimental results of repolarized ceramics showed that a high level of poling is retained. On the other hand, Lee et al. [18] studied the effect of the residual stress induced during the heat-treatment process on the properties of PZT films. They showed that the remnant and saturation polarizations increase when a compressive stress is induced. Although the effect of temperature on the piezoelectric behavior of PZT ceramics has been investigated, systematic and fundamental studies on the thermally induced electromechanical behavior of the piezoelectric composite materials are limited [19,20] and more work is needed.

The finite element method (FEM) is regarded as an important tool for solving problems of engineering and mathematical methods. Some finite element works on the modeling and design of the piezoelectric composite materials have been presented [21,22,23]. By using the FEM, we can easily obtain the electromechanical response for piezoelectric composite materials. 

In this paper, we investigated the electromechanical response of metal-core piezoelectric fiber-reinforced Al matrix (metal-core piezoelectric fiber/Al) composite material subjected to thermal and mechanical loading. Three-dimensional finite element simulations of the metal-core piezoelectric fiber/Al matrix composite material were conducted, and the residual thermal stress generated during the solidification process of the Al matrix was predicted. The piezoelectricity of this composite was characterized by FEM, and then the output voltage of the composite under concentrated load was calculated in consideration of its use as a load position detection sensor. In addition, the numerical and measured values of the residual stress were compared.

## 2. Analytical Procedure

### 2.1. Basic Equations

Considering a piezoelectric material with no body force and free charge. The field Equations in the Cartesian coordinates *x_i_* (*i* = 1, 2, 3) were given by

(1) $$\sigma_{ji,j}=\;0$$

(2) $$D_{i,i}=0$$

where *σ_ij_* and *D_i_* are the components of the stress tensor and electric displacement vector, respectively, a comma followed by an index denotes partial differentiation with respect to the space coordinates *x_i_*, and the Einstein summation convention over repeated indices was used. The relation between the strain tensor component *ε_ij_* and the displacement vector component *u_i_* is given by

(3) $$\varepsilon_{ij}=\frac{1}{2}(u_{j,i}+u_{i,j})$$

and the electric field intensity vector component *E_i_* is:

(4) $$E_{i}=-\phi ,i$$

where *ϕ* is the electric potential. Constitutive relations can be written as

(5) $$\varepsilon_{ij}=s_{ijkl}\sigma_{kl}+d_{kij}E_{k}+\alpha_{ij}\Delta T_{\;}$$

(6) $$D_{i}=d_{ikl}\sigma_{kl}+\varepsilon_{ik}^{T}E_{k}\;+p_{i}\Delta T$$

where *T* is the temperature, $s_{ijkl}$, $d_{kij}$, $\;\varepsilon_{ik}^{T}\;$ are the elastic compliance, piezoelectric coefficient, and dielectric permittivity at constant stress, respectively, and $\alpha_{ij}^{}$ is the CTE, which satisfy the following symmetry relations:

(7) $$s_{ijkl}=\;s_{jikl}=\;s_{ijlk}=\;s_{klij},d_{kij}=d_{kji},{є}_{ik}^{T}={є}_{ki}^{T},\alpha_{ij}^{}=\alpha_{ji}^{}$$

In Equation (6), *p_i_* is the pyroelectric constant. The constitutive Equations (5) and (6) for piezoelectric material poled in the *x*_3_ direction are:

(8) $$\left\{\begin{array}{c} \varepsilon_{11}\\ \varepsilon_{22}\\ \varepsilon_{33}\\ 2\varepsilon_{23}\\ 2\varepsilon_{31}\\ 2\varepsilon_{12} \end{array}\right\}=\left[\begin{array}{cccccc} s_{11} & s_{12} & s_{13} & 0 & 0 & 0\\ s_{12} & s_{11} & s_{13} & 0 & 0 & 0\\ s_{13} & s_{13} & s_{33} & 0 & 0 & 0\\ 0 & 0 & 0 & s_{44} & 0 & 0\\ 0 & 0 & 0 & 0 & s_{44} & 0\\ 0 & 0 & 0 & 0 & 0 & s_{66} \end{array}\right]\left\{\begin{array}{c} \sigma_{11}\\ \sigma_{22}\\ \sigma_{33}\\ \sigma_{23}\\ \sigma_{31}\\ \sigma_{12} \end{array}\right\}+\left[\begin{array}{ccc} 0 & 0 & d_{31}\\ 0 & 0 & d_{31}\\ 0 & 0 & d_{33}\\ 0 & d_{15} & 0\\ d_{15} & 0 & 0\\ 0 & 0 & 0 \end{array}\right]\left\{\begin{array}{c} E_{1}\\ E_{2}\\ E_{3} \end{array}\right\}+\left[\begin{array}{c} \alpha_{33}\\ \alpha_{33}\\ \alpha_{33}\\ 0\\ 0\\ 0 \end{array}\right]\Delta T$$

(9) $$\left\{\begin{array}{c} D_{1}\\ D_{2}\\ D_{3} \end{array}\right\}=\left[\begin{array}{cccccc} 0 & 0 & 0 & 0 & d_{15} & 0\\ 0 & 0 & 0 & d_{15} & 0 & 0\\ d_{31} & d_{31} & d_{33} & 0 & 0 & 0 \end{array}\right]\left\{\begin{array}{c} \sigma_{11}\\ \sigma_{22}\\ \sigma_{33}\\ \sigma_{23}\\ \sigma_{31}\\ \sigma_{12} \end{array}\right\}+\left[\begin{array}{ccc} {є}_{11}^{T} & 0 & 0\\ 0 & {є}_{11}^{T} & 0\\ 0 & 0 & {є}_{33}^{T} \end{array}\right]\left\{\begin{array}{c} E_{1}\\ E_{2}\\ E_{3} \end{array}\right\}+\left[\begin{array}{c} p_{3}\\ p_{3}\\ p_{3} \end{array}\right]\Delta T$$

where:

(10) $$\sigma_{23}=\sigma_{32},\begin{array}{cc} & \sigma_{31}=\sigma_{13}, \end{array}\begin{array}{cc} & \begin{array}{cc} \sigma_{12}=\sigma_{21} & \end{array} \end{array}$$

(11) $$\varepsilon_{23}=\varepsilon_{32},\begin{array}{cc} & \varepsilon_{31}=\varepsilon_{13}, \end{array}\begin{array}{cc} & \begin{array}{cc} \varepsilon_{12}=\varepsilon_{21} & \end{array} \end{array}$$

(12) $$\begin{array}{l} s_{11}=s_{1111}=s_{2222},\begin{array}{cc} & s_{12}=s_{1122}, \end{array}\begin{array}{cc} & s_{13}=s_{1133}=s_{2233}, \end{array}\begin{array}{cc} & s_{33}=s_{3333}, \end{array}\\ s_{44}=4s_{2323}=4s_{3131},\begin{array}{cc} & s_{66}=4s_{1212}=2\left(s_{11}-s_{12}\right) \end{array} \end{array}$$

(13) $$d_{15}=2d_{131}=2d_{223},\begin{array}{cc} & d_{31}=d_{311}=d_{322}, \end{array}\begin{array}{cc} & d_{33}=d_{333} \end{array}$$

### 2.2. Model

**Figure 1:**
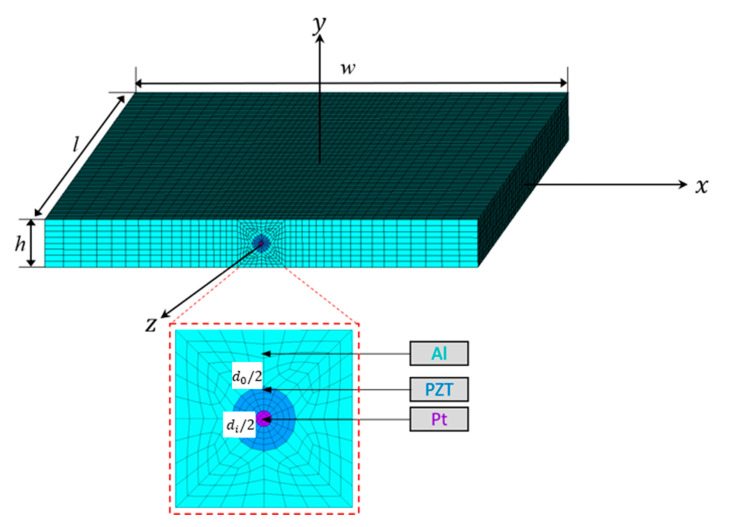
Finite element analysis model of lead zirconate titanate (PZT) piezoelectric fiber/Al composite.

The finite element analysis software, ANSYS^®^, was used in this investigation. A three-dimensional finite element model was necessary to accurately present the electromechanical field distribution of the metal-core piezoelectric fiber/Al matrix composite material. Figure 1 shows the finite element model. A rectangular Cartesian coordinate system (*x*, *y*, *z*) is used for modeling with coincidence between the *z* axis and the direction of the piezoelectric fiber. Meanwhile, the origin of the coordinate system coincides with the center of the composite material. This model consists of a platinum (Pt) fiber (diameter *d*_i_, length *l*) embedded into a circular PZT tube (outer diameter *d*_o_, inner diameter *d*_i_ and length *l*) and an Al plate (length *l*, width *w*, and thickness *h*). The problem was also formulated using a cylindrical coordinate system (*r*, *θ*, *z*), where the longitudinal axis of the Pt core also coincides with the *z* axis. We assumed that the Curie temperature of the PZT fiber was 127 °C. Hence, the direction of each electric domain in PZT was randomly oriented [24], and the piezoelectric coefficients were zero above the Curie temperature.

**Figure 2:**
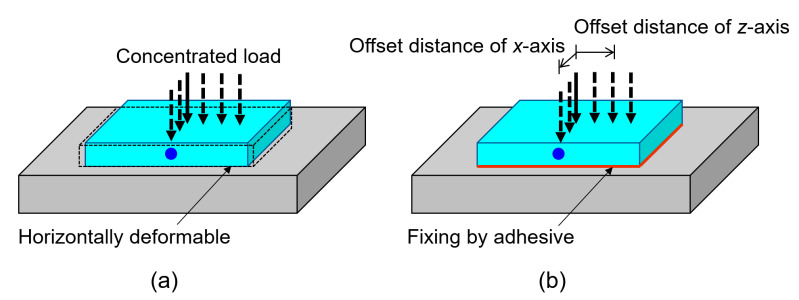
Illustration of the model with (**a**) center point fixed and (**b**) whole surface fixed.

We considered the following thermal and mechanical loadings of the piezoelectric fiber/Al matrix composite material: (i) the composite was cooled from high temperature *T* = 660 °C (melting point of pure Al), 600 °C or 400 °C to 20 °C (room temperature) and (ii) after cooling, we considered the mechanical loading, i.e., the composite was under tensile stress *σ_zz_* = *σ*_0_ at *z* = *l*/2 and the displacement *u_z_* = 0 at *z* = −*l*/2. In addition, in order to examine the performance of the load position detection sensor of this metal-core piezoelectric fiber/Al matrix composite material, we applied the concentrated load of 1 N at various locations on the top surface (*y* = *h*/2). At this time, it was assumed that the bottom surface had two types, that is, the case where the center point is fixed (*u_x_* = *u_y_* = *u_z_* = 0 at *x* = 0, *y* = −*h*/2, *z* = 0) and the case where the whole surface is fixed (*u_x_* = *u_y_* = *u_z_* = 0 at −*w*/2 ≤ *x* ≤ *w*/2, *y* = −*h*/2, −*l*/2 ≤ *z* ≤ *l*/2). Here, the model with the center point fixed assumes the composite material placed on a rigid floor (this support is horizontally deformable as shown in Figure 2a), and the model with the whole surface fixed shows the composite material bonded to a rigid floor (Figure 2b). 

Table 1 lists the material properties of PZT used for each domain that is randomly distributed. For simplicity, the pyroelectric constant was ignored here. Table 2 lists the Young’s modulus *E*, Poisson’s ratio *ν* and the CTE of Al and Pt. The length, width, and thickness of the composite are *l* = 20 mm, *w* = 5 mm, and *h* = 0.55 mm. The outer diameter and inner diameter of the PZT tube are *d*_o_ = 0.2 mm and *d*_i_ = 0.05 mm, respectively. 

**Figure 3:**
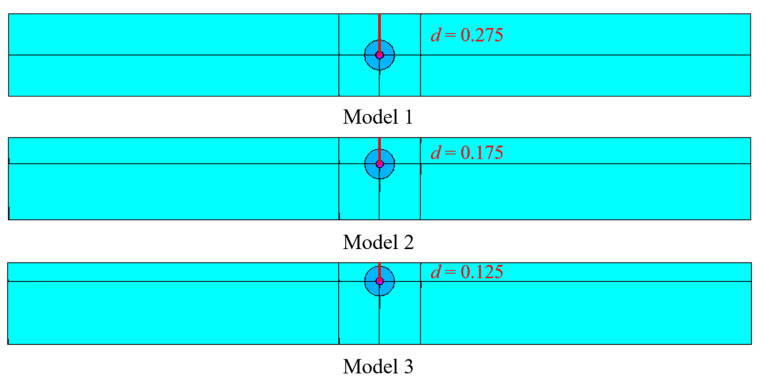
Planes perpendicular to the *z* axis of three models.

In addition, the distance *d* from the center of the Pt to one side of the *y* axis was changed to locate the optimal position of the PZT fiber as shown in Figure 3. The distances from the plate surface to the fiber center for Models 1, 2 and 3 were *d* = 0.275, 0.175 and 0.125 mm, respectively.

## 3. Experimental Procedure

Details of the preparation of the sample can be found in Ref. 15. Metal-core piezoelectric fiber, Al plate with a U-shaped groove on which a copper (Cu) foil is placed on the surface, and the Al plate were prepared. After placing the metal-core piezoelectric fiber into the groove, another Al plate was placed over the fiber and Cu foil, and hot pressing was performed at 600 °C. After hot pressing, the composite sample was cut off. 

**Figure 4:**
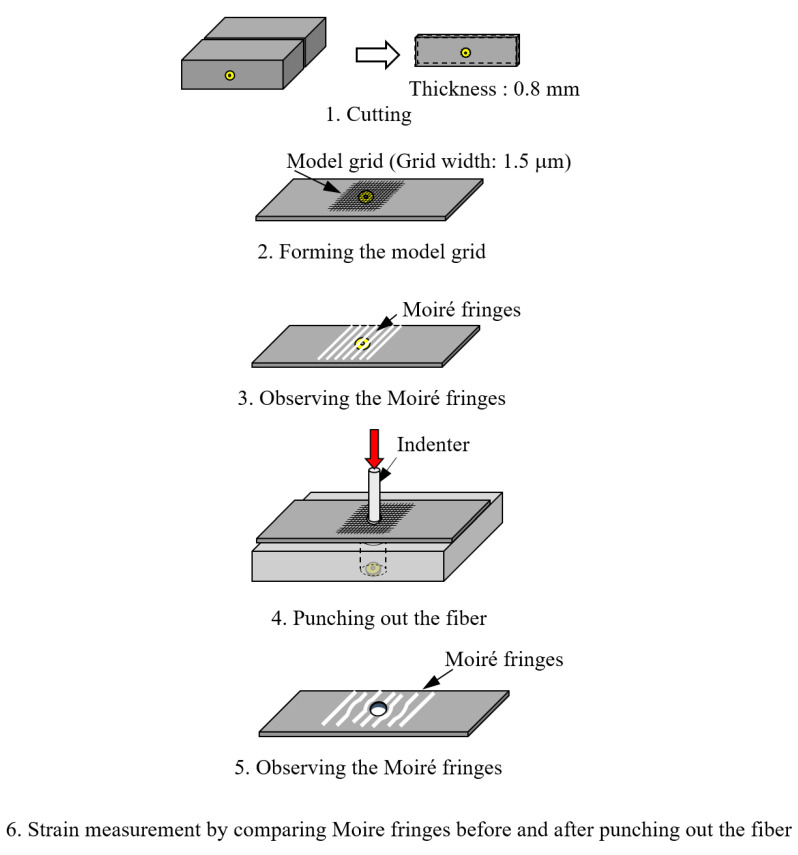
Experimental procedure of residual stress measurement by electron beam Moiré method.

The compressive residual stress generated in the metal-core piezoelectric fiber/aluminum composite was measured by the electron beam Moiré method [25]. Using this method, the microscopic deformation of Al when the metal core piezoelectric fiber was removed was measured, and the residual stress was determined from the deformation (Figure 4).

A metal-core piezoelectric fiber/Al composite was cut with a diamond cutter (IsoMet Low Speed, Buehler Corp. Lake Bluff, IL, USA) to prepare a 0.8 mm-thick specimen. An electron beam resist (ZEP520A, Zeon Co., Ltd. Tokyo, Japan) was uniformly applied to the polished specimen surface with a spin coater (1H-D7, Misaka Inc. Tokyo, Japan), and it was dried at 50 °C for 3.6 ks using a drying oven (FO-30WT, Tokyo Garasu Kikai Co., Ltd. Tokyo, Japan). The coated resistance was exposed in a grid pattern by scanning an electron beam with a scanning electron microscope (SEM, SX-40A, Topcon Corp. Tokyo, Japan) at intervals of 1.5 μm, and the resistance in the portion irradiated with the electron beam was removed by a developer (ZED-N50, Zeon Co., Ltd. Tokyo, Japan). A model grid was formed by depositing gold onto the removed portion using a sputtering device (JFC-1500, JEOL Ltd. Tokyo, Japan). After this, the Moiré fringes generated using an irradiating electron beam as a master grid for this model grid were observed by SEM.

Subsequently, the specimen was fixed to a jig and the piezoelectric fiber was punched out of the Al with an indenter with a diameter of 0.15 mm. After this, the Moiré fringes were observed by the same method as described above. The residual stress was calculated from the strain that was measured by comparing the Moiré fringes before and after punching out the fiber.

## 4. Results and Discussion

### 4.1. Analytical Results

**Figure 5:**
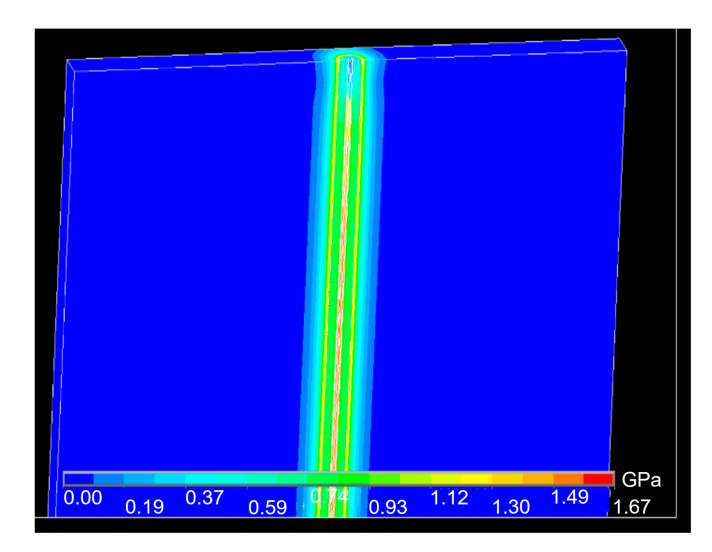
Distribution of the compressive residual thermal stress after the temperature decreases from the melting point to room temperature.

**Figure 6:**
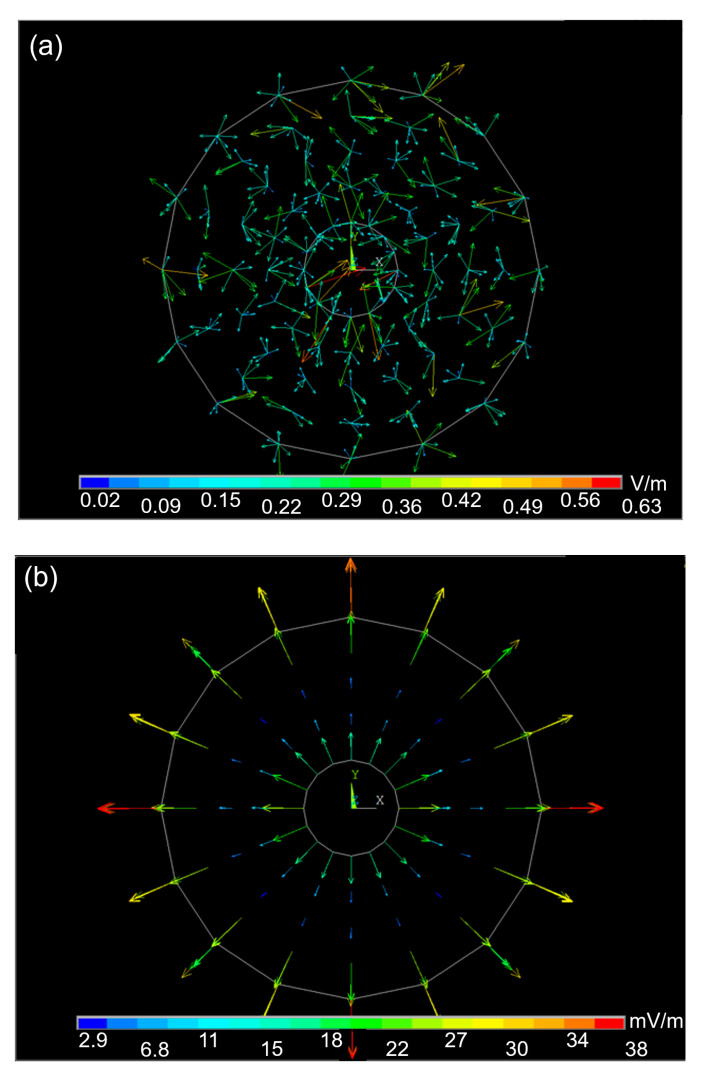
Direction of the electric field (**a**) before and (**b**) after the temperature decreases from the melting point to room temperature.

To simulate the Al solidification process, the reference temperature was defined as the melting point of Al (660 °C) for thermal stress calculations. Figure 5 shows the distribution of the thermal stress of the piezoelectric fiber/Al matrix composite after the Al solidified. High compressive residual stress can be observed at the interface between the PZT fiber and the Al matrix. After the piezoelectric composite was manufactured, electrical poling was necessary for achieving piezoelectricity. Figure 6a shows the initial direction of the electric field of the PZT fiber. Figure 6b demonstrates the change in the direction of the electric field, after the temperature decreases from the melting point to room temperature. This indicates that when the temperature decreased from high temperature to room temperature, a large residual stress was generated, and as a result, an electric field was generated and polarized. In the polarization process, the Pt was used as the electrode, hence the direction of polarization was radial. 

**Figure 7:**
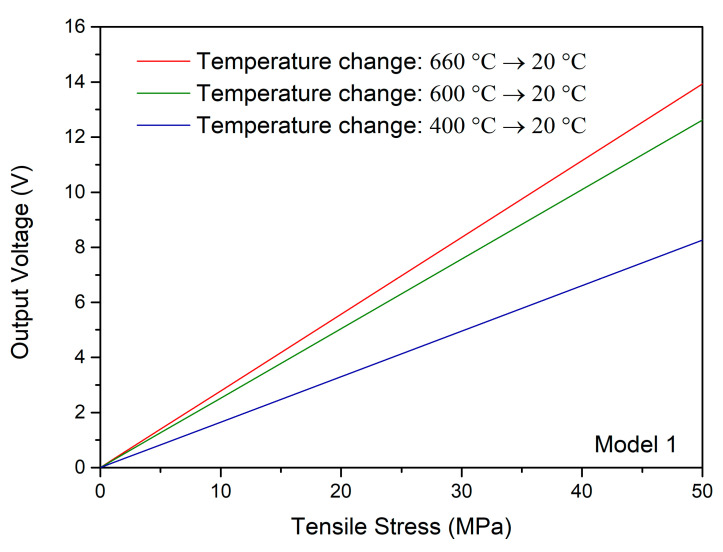
Output voltages versus applied tensile stress for the composite (Model 1) after the temperature decreases from 660 °C (melting point of pure Al), 600 °C or 400 °C to 20 °C.

Then, the piezoelectricity was characterized by applying tensile stress to calculate the output voltage response. Under the residual stress and applied tensile stress *σ*_0_ = 1 MPa, the output voltage *V*_out_ = 1.11 V was obtained. We also examined the piezoelectric properties of the piezoelectric fiber/Al matrix composite under the residual stress in detail. The output voltage was recorded when different residual stresses were considered, and all boundary conditions were set as the same. Figure 7 indicates the output voltage of different residual stress situations versus applied tensile stress. We can clearly see that output voltages are increased according to the additions of tensile stresses, and the relationship between output voltage and applied tensile stress is linear. Nevertheless, when residual stress is added, the output voltage is larger, and the increment of output voltage is also larger. This is because when this composite was subjected to tensile stress, the strain was increased owing to the high compressive residual stress, so that the piezoelectricity of this composite can be improved. This situation may be applicable to the other piezoelectric composites with a different fiber size, shape, and matrix material. The results for the evaluation of output voltage and residual stress may help piezoelectric composite designers to optimize performance.

**Figure 8:**
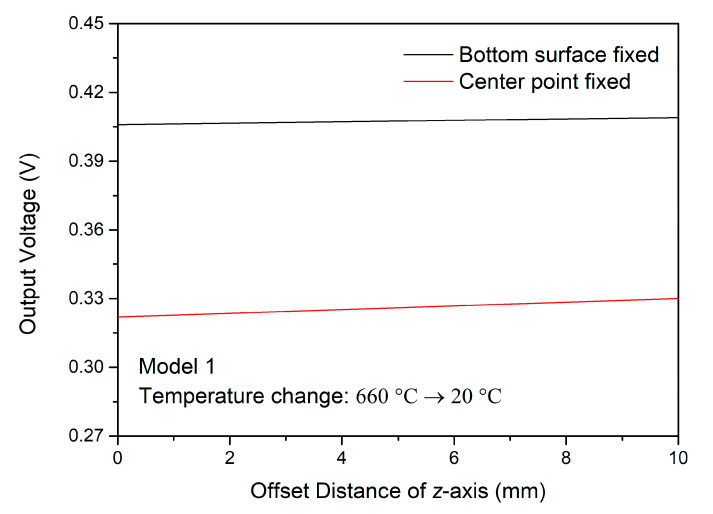
Relationships between the output voltage for the composite (Model 1) due to the concentrated load and the offset distance along the *z* axis (*x* = 0 mm).

**Figure 9:**
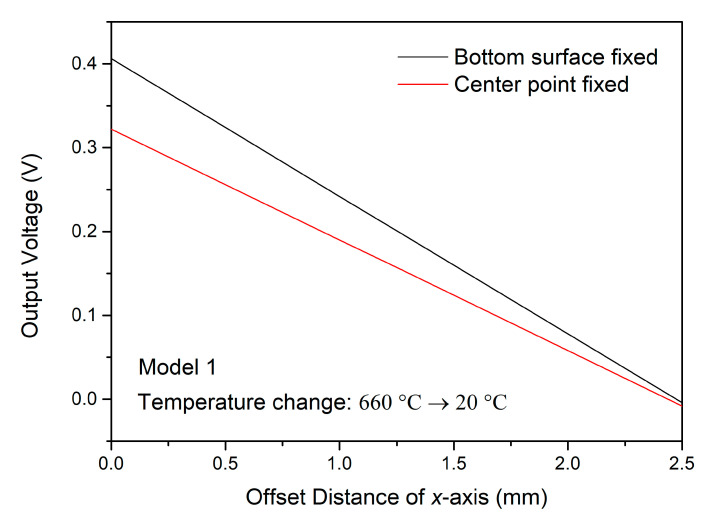
Relationships between the output voltage for the composite (Model 1) due to the concentrated load and the offset distance along the *x* axis (*z* = 0 mm).

Instead of the usual use as an energy-harvesting device (e.g., piezoelectric vibration energy harvester [26,27]), we tried to utilize this composite as a load position detection sensor, and its performance was discussed. We assumed that this composite was used for this application by fixing the central point of the bottom surface (Figure 2a) or settling all points of the bottom surface (Figure 2b) as an embedded sensor. Through checking the output voltage under the same magnitude of concentrated load but different applied position, the relationship between output voltage and offset distance can be clarified. As a result of the symmetry, the center of the top surface was set as the initial point. The results of the output voltage and offset distance along the *z* axis and *x* axis were shown in Figure 8 and Figure 9, respectively. For the *z* axis, the output voltage variation changing the *z* coordinate value of the force point (point at which the force is applied) was very small. Nevertheless, the output voltage change along the *x* axis is conspicuous, and as the offset distance along the *x* axis increases, the output voltage decreases. From these results, we can predict the offset distance along the *x* axis based on the output voltage. When detecting the offset distance along the *z* axis, it is necessary to arrange the two PZT fibers in a cross shape. It was also shown that when used as the load position detection sensor, the performance is better if all points of the bottom surface of the composite are fixed.

**Figure 10:**
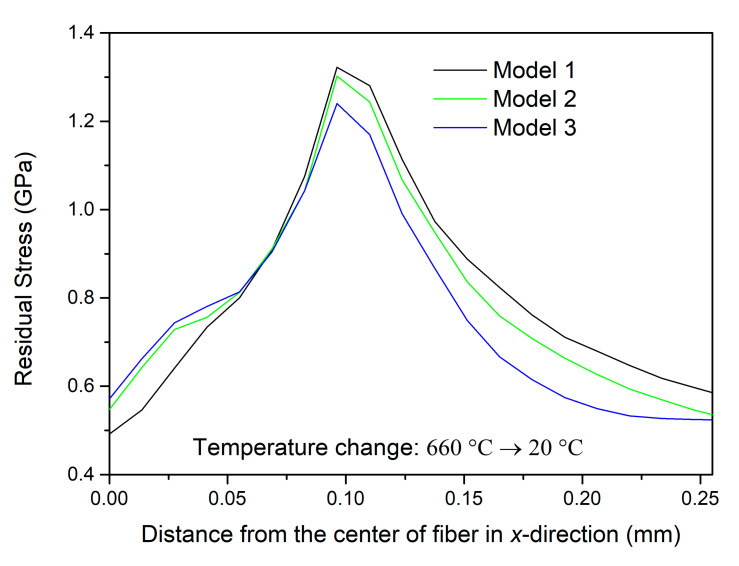
Compressive residual stress distribution for the composites (Model 1, 2 and 3) along the *x* direction at *y* = *z* = 0 mm.

Furthermore, the relationship between the compressive residual stress *σ_xx_* and distance *d* for the three models are also shown in Figure 10. The residual stresses of the three models all reached the peak value in the interface of the PZT fiber and Al matrix first, then decreased. Simultaneously, the residual stresses of Model 1 were larger than the other two models due to the symmetry. The higher the compressive residual stress, the better the piezoelectricity of the composite (see Figure 7). Therefore, it is desirable to embed the PZT fiber in the center of the Al plate as much as possible.

### 4.2. Experimental Results

**Figure 11:**
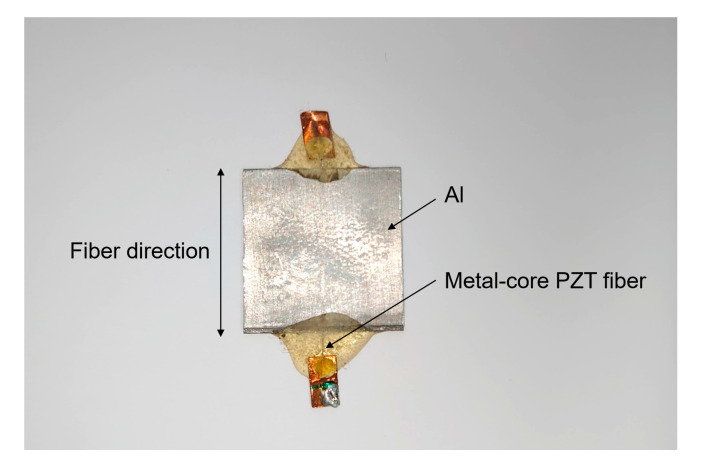
Metal-core lead zirconate titanate (PZT) piezoelectric fiber/Al composite sample.

**Figure 12:**
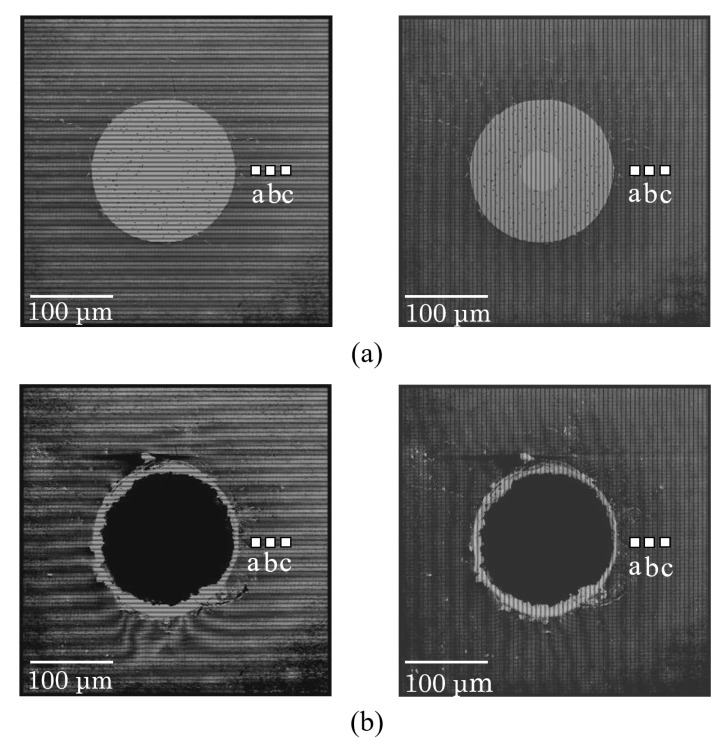
Difference in the Moiré patterns (**a**) before and (**b**) after metal-core piezoelectric fiber indentation.

**Figure 13:**
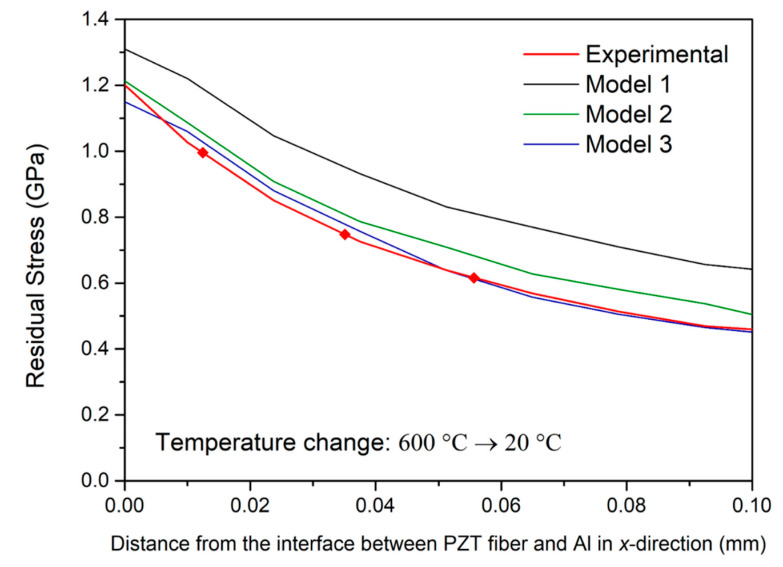
Compressive residual stress distribution along the *x* direction at *y* = *z* = 0 mm of three models and the experiment.

Figure 11 shows a metal-core piezoelectric fiber/Al composite sample. Figure 12 shows the SEM images the Moiré patterns (a) before and (b) after metal-core piezoelectric fiber indentation. The right side of the photos is the Moiré pattern in the *x* direction, and the left is the one in the *y* direction. The obtained strains in the *x* and *y* directions are converted into the stress in the radial direction. Figure 13 shows the measured values (red dot) of the compressive residual stress and its approximate curve (red line) for the metal-core piezoelectric fiber/Al composite sample. The calculated values of the three models after the temperature decreased from 600 °C to 20 °C are also shown. The experimental value falls between Model 2 and Model 3; considering that the experimental error may occur during the manufacturing process, it can be inferred that the theoretical value agrees with the experimental value. 

## 5. Conclusions

Numerical and experimental approaches were presented to characterize the electromechanical response of metal-core piezoelectric fiber/Al composite subjected to thermal and mechanical loading. It was found that the residual stress dramatically increases the piezoelectricity of the metal-core piezoelectric fiber/Al composites. It was also shown that this composite is useful as a load position sensor. The results of this study will help to offer a basis for optimizing piezoelectric composite performance by selecting the optimal residual stress and structure.

## Figures and Tables

**Table 1:** Material properties of PZT.

Elastic compliance (×10^−12^ m^2^/N)	
*s*_11_	16.5
*s*_33_	20.7
*s*_44_	43.5
*s*_12_	−4.78
*s*_13_	−8.45
Piezoelectric coefficient (×10^−12^ m/V)	
*d*_31_	−274
*d*_33_	593
*d*_15_	741
Permittivity (×10^−10^ C/Vm)	
${є}_{11}^{T}$	277
${є}_{33}^{T}$	301
CTE (×10^−6^ /K)	
$\alpha_{33}$	7.5

**Table 2:** Material properties of Al and Pt.

	**Al**	**Pt**
Young’s modulus (×10^9^ N/m^2^)		
*E*	69	168
Poisson’s ratio		
ν	0.34	0.38
CTE (×10^−6^ /K)		
α	23.9	8.9
